# Test characteristics of common appendicitis scores with and without laboratory investigations: a prospective observational study

**DOI:** 10.1186/s12887-016-0687-6

**Published:** 2016-08-30

**Authors:** Ijab Khanafer, Dori-Ann Martin, Tatum P. Mitra, Robin Eccles, Mary E. Brindle, Alberto Nettel-Aguirre, Graham C. Thompson

**Affiliations:** 1 Department of Pediatrics, University of Calgary, Calgary, AB Canada; 2 Faculty of Kinesiology, University of Calgary, Calgary, AB Canada; 3 Department of Surgery, University of Calgary, Calgary, AB Canada; 4 Department of Community Health Sciences, University of Calgary, Calgary, AB Canada; 5 Department of Emergency Medicine, University of Calgary, Calgary, AB Canada

**Keywords:** Appendicitis, Child, Leukocyte count, Neutrophils, Referral and consultation

## Abstract

**Background:**

Despite the poor independent test characteristics of the white blood cell count (WBC) and neutrophil count (NC) in identifying appendicitis, common clinical decision supports including the Pediatric Appendicitis Score (PAS) and Alvarado Score (AS), require the WBC and NC values. Moreover, blood tests cause discomfort/pain to children and require time for processing results. Scores based on clinical information alone may be of benefit in the pediatric population. The objective of our study was to determine the test characteristics of the PAS and the AS with and without laboratory investigations (mPAS, mAS respectively) as well as the Lintula Score.

**Methods:**

A prospective cohort study of children aged 5–17 years presenting to a pediatric ED with suspected appendicitis. Clinical care of the patient was left to the managing physician. At risk for appendicitis was defined by PAS ≥6; AS ≥5; LS ≥16, as originally described; modified cutoffs were defined as mPAS ≥4; mAS ≥4. Appendicitis was defined as acute inflammation, rupture or abscess of the appendix on pathologic evaluation. Test characteristics for each of the 5 scores were calculated.

**Results:**

Of the 180 eligible children, 102 (56.7 %) were female. The average age was 11.2 years (SD 3.1). Appendectomy was performed in 58 (32.2 %) of children, 55 (94.8 %) were positive. For the PAS and mPAS, sensitivity and negative predictive values were similar (80.0 %, 86.4 % vs 87.3 %, 85.1 % respectively). For the AS and mAS, sensitivity and negative predictive values were also similar (85.5 %, 87.1 % vs 83.6 %, 83.3 % respectively). Specificities in the PAS, mPAS, AS and mAS were low (56.0 %, 32.0 %, 43.2 %, 63.0 % respectively). Test characteristics of the LS were poor (59.3 %, 79.2 %, 55.2 %, 81.8 %).

**Conclusions:**

A modified Alvarado and PAS can be used to screen for children at low risk of appendicitis who may be carefully observed at home without the need for laboratory investigation. Translation to primary care settings should evaluate generalizability and determine impact on referral patterns.

**Electronic supplementary material:**

The online version of this article (doi:10.1186/s12887-016-0687-6) contains supplementary material, which is available to authorized users.

## Background

Appendicitis is the most common non-traumatic surgical emergency in the pediatric population [1], affecting an estimated 80,000 children in the United States annually, at a rate of 4 per 1,000 children under the age 14 years [2]. Early diagnosis may decrease risk of progression to perforation, abscess formation and sepsis, which are major causes of childhood morbidity [2]. Despite its high incidence, diagnosing appendicitis can be difficult due to the non-specific or atypical nature of its symptoms [3]. Numerous scoring systems, such as the Alvarado score (AS) [4], the Pediatric Appendicitis Score (PAS) [5] and Lintula score (LS) [6] (Table 1) have been developed in an attempt to assist clinicians in recognizing which children presenting with abdominal pain are at greatest risk of having appendicitis. These clinical scores are based on elements of history and physical exam, with the vast majority of scores incorporating basic laboratory investigations including the White Blood Cell (WBC) and Neutrophil counts (NC).

**Table 1:** Alvarado score, Pediatric appendicitis score and Lintula score

	Alvarado score [4]		Pediatric appendicitis score [5]		Lintula score [6]	
	Migration to right lower quadrant	1	Migration of pain	1	Male	2
	Anorexia - acetone	1	Anorexia	1	Intensity of pain = severe	2
	Nausea - vomiting	1	Nausea/emesis	1	Relocation of pain	4
	Tenderness in the right lower quadrant	2	Tenderness over the right iliac fossa	2	Vomiting	2
	Rebound pain	1	Cough/percussion/hopping tenderness in the right lower quadrant	2	Pain in right lower quadrant	4
	Elevation of temperature > 37.3	1	Pyrexia	1	Fever >37.5	3
	Leukocytosis >10.0 × 10 ^9^ /L	2	Leukocytosis > 10.0 × 10 ^9^ /L	1	Guarding	4
	Shift to the left >75 %	1	Polymorphonuclear neutrophilia	1	Absent, tinkling or high-pitched bowel sounds	4
					Rebound tenderness	7
Total	10		10		32	
Score interpretation	Non-appendicitis	<5	Non-appendicitis	≤5	Low probability of appendicitis – amenable to discharge	≤15
	Compatible with appendicitis – may be observed	5–6	High probability of appendicitis	≥6	Intermediate probability of appendicitis – further observation	16–20
	Probable appendicitis – requires surgery	7–8			High probability of appendicitis – emergency surgery	≥21
	Very probable appendicitis – requires surgery	9–10				

Components based on laboratory investigation have been underlined

The use of the WBC count in the diagnosis of acute appendicitis is subject to several limitations. First, children with abdominal pain often first present to care to a primary care provider or walk-in clinic where laboratory resources may not available. From the patient perspective, bloodwork causes pain, distress, as well as anxiety [7–9]. In addition, the time required for completion of the WBC and NC may increase time to diagnosis and surgical consultation. Moreover, routine performance of these tests may lead to unwarranted health care costs. Finally, the reported sensitivities and specificities of the WBC and NC range from 60 to 100 % [10–12] and 20–53 % [11, 12] respectively. Given the aforementioned limitations of the WBC and NC for the diagnosis of appendicitis, scores relying exclusively on clinical signs and symptoms may be of benefit. Therefore, the aim of this study was to determine the sensitivity, specificity and predictive values (test characteristics) of the Alvarado Score, Pediatric Appendicitis Score and the Lintula Score in a pediatric emergency department (ED) setting, when calculated exclusively on clinical features.

## Methods

### Study design, population and setting

We performed a prospective cohort study of children presenting to the Alberta Children’s Hospital ED with suspected appendicitis. Our hospital, located in Calgary, Alberta, Canada is the tertiary care referral centre for southern Alberta, western Saskatchewan and eastern British Columbia. It has a catchment size of approximately 1.8 million patients. The ED provides care to approximately 72,000 patients annually.

Between February 26, 2013 and January 5, 2014, we enrolled children between the ages of 5 and 17 years who a) presented to ACH ED with complaints of abdominal pain for less than 5 days and b) had appendicitis in their differential diagnosis as per the managing ED team. In order to be included in the study, a WBC had to have been ordered. Furthermore, study subjects had to be evaluated by a senior pediatric resident, a Pediatric Emergency Medicine (PEM) fellow or a PEM staff physician.

We excluded patients with abdominal pain for whom appendicitis was not in the differential diagnosis, patients with previous appendectomy or other abdominal surgery, patients with imaging studies that were positive for appendicitis prior to presentation to our hospital, as well as patients who were pregnant, had immunosuppressive disorders, were non-verbal or whose family was unable to complete the consent form due to a language barrier. Children with chronic gastrointestinal comorbidities were not excluded, but comorbidities were recorded as potential confounders.

### Study process

This study was approved by the University of Calgary Conjoint Health Research Ethics Board. All investigators/authors decline competing interests. Patients were recruited and enrolled between the hours of 8 AM and midnight by trained Pediatric Emergency Medicine Research Associate Program (PEMRAP) team members. Consent from guardians, and assent from patients 7 years or older, were obtained. Following enrolment, PEMRAP members completed case report forms regarding the course of the presenting illness including history and duration of nausea, vomiting, anorexia and fever. The evaluating PEM physician completed a case report form with elements of the physical exam prior to reviewing any results of imaging, blood work or surgical consult. All data was collected on standardized case report forms developed specifically for study use. These forms grouped elements of the history and physical exam separately, while elements within the groups were presented in random order.

Using the PEMRAP and clinician case report forms and laboratory data, scores were calculated for the AS, modified AS (mAS), PAS, modified PAS (mPAS) and LS. mAS and mPAS were derived by simply removing the WBC and NC component of the original scores (Table 2). Additional study data, including demographics, ED, surgical and inpatient management, were captured through Health Records review. To detect return visits to any acute care centre within the region, those who were discharged home were followed for 2 weeks using provincial electronic administrative databases. Data management was locally performed using REDCap [13], a secure web-based application designed to support data capture for research studies.

**Table 2:** Modification of the Alvarado Score and Pediatric Appendicitis Score by removal of laboratory investigations

Modified Alvarado score		Modified Pediatric appendicitis score	
Migration to right lower quadrant	1	Migration of pain	1
Anorexia - acetone	1	Anorexia	1
Nausea - vomiting	1	Nausea/emesis	1
Tenderness in the right lower quadrant	2	Tenderness over the right iliac fossa	2
Rebound pain	1	Cough/percussion/hopping tenderness in the right lower quadrant	2
Elevation of temperature > 37.3	1	Pyrexia	1
Total	7		8

Throughout the course of the study, clinical care of the patient was left to the discretion of the managing physician. Managing physicians were not made aware of study-generated appendicitis scores.

### Outcomes

The primary patient outcome of interest was the presence of appendicitis, defined as the presence of acute inflammation, rupture or abscess of the appendix on pathologic evaluation. The primary analyses of interest were the sensitivity and negative predictive value of the appendicitis scores (mAS, AS, mPAS, PAS, Lintula). Secondary analyses of interest were the specificity, positive predictive value and accuracy of the appendicitis tests.

### Definitions

Because pyrexia and neutrophilia were not specifically defined in the original PAS manuscript by Samuel [5], we defined pyrexia as temperature > 37.5C and neutrophilia as a differential showing >75 % neutrophils. The definitions used for the AS and LS were those described in their respective derivation manuscripts [4, 6].

### Statistical analysis

A sample size of 126 patients was calculated to achieve a margin of error of at most 8 % for sensitivity with 95 % confidence interval, assuming an existing sensitivity of 70 %, as at the time of study design this is approximately the lowest sensitivity found for the Alvarado Score, the Pediatric Appendicitis Score and the Lintula Score in the literature (Additional file 1: Table S1) [1, 4, 5, 14–19]. We calculated Receiver Operating Characteristics (ROC) using STATA (STATA SE v12.1 Station College, TX), in order to determine the sensitivity, specificity, predictive values and accuracy for the Alvarado Score, the Pediatric Appendicitis Score, their modified counterparts, as well as the Lintula Score. We defined an absolute decrease in screening tool test characteristics (with vs without laboratory investigation) of ≥5 % as having clinical significance. While all test characteristics were calculated, we specifically identified sensitivity (to optimize capture of patients with appendicitis) and Negative Predictive Value (NPV - to be ensure those identified as negative were truly negative) as target test characteristics. Furthermore, Cohen’s Kappa was calculated to measure agreement between the cut-offs used in the original scores and the cut-offs we are proposing for the modified scores.

## Results

**Fig. 1:**
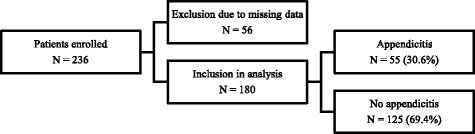
Patients enrolled, excluded, analyzed

We enrolled 236 children, of which 56 were excluded from analysis due to missing data reflecting any single element of the scoring systems, making it impossible to calculate their appendicitis scores. A complete set of data from a total of 180 children was analyzed (Fig. 1). The average age of the study population was 11.2 years (SD 3.1); 56.7 % (102) were female. Appendectomy was performed in 58 (32.2 %) children. The negative appendectomy rate was 5.2 % (3/58). Age, previous health care visits and presence of gastrointestinal co-morbidities were similar between children with and without appendicitis; however, there was a higher proportion of females in the group without appendicitis (78, 62.4 % vs 24, 43.6 %, Table 3). Ultrasound was performed in the vast majority of cases (164, 91.1 %), with only 9 (5.0 %) of children having computed tomography (CT) imaging.

**Table 3:** Demographics of children presenting with suspected appendicitis

	No Appendicitis (*n* = 125)	Appendicitis (*n* = 55)	Overall (*n* = 180)	*p*-value
Age, years - Mean (SD)	11.2 (3.3)	11.2 (2.8)	11.2 (3.1)	0.96
Female - n (%)	78 (62.4)	24 (43.6)	102 (56.7)	0.02
Previous HCP Visit for same illness - n (%)	32 (25.6)	15 (27.3)	47 (26.2)	0.79
Previous ED visit for same illness - n (%)	14 (11.2)	10 (18.2)	24 (13.3)	0.20
GI comorbidity - n (%)	2 (1.6)	1 (1.8)	3 (1.7)	0.99
US performed - n (%)	113 (90.4)	51 (92.7)	164 (91.1)	0.62
CT performed - n (%)	8 (6.4)	1 (1.8)	9 (5.0)	0.28

*CT* Computed Tomography, *ED* Emergency Department, *GI* Gastrointestinal, *HCP* Health Care Provider, *US* Ultrasound

**Fig. 2:**
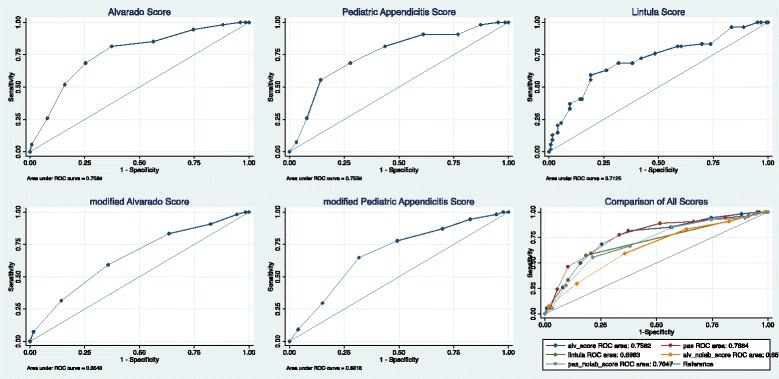
Receiver Operating Characteristic curve for appendicitis scores with and without laboratory investigations

Table 4 demonstrates the results for our primary objective, the test characteristics of the AS and PAS with and without laboratory investigations (mAS, mPAS). For the mAS, a cutoff value of 4 resulted in sensitivity and NPV closest to the AS cutoff of 5, as originally described by Alvarado. Similarly, a cutoff value of 4 for the mPAS most closely approximated the original PAS cutoff of 6. Figure 2 outlines the receiver operating curves for the appendicitis scores.

**Table 4:** Test Characteristics of AS, mAS, PAS, mPAS

AS	Score	Sensitivity	Specificity	PPV	NPV	Accuracy	mAS	Score	Sensitivity	Specificity	PPV	NPV	Accuracy
	1	100.0	1.6	30.9	100.0	31.1		1	100.0	1.6	30.9	100	31.1
	2	100.0	4.0	31.4	100.0	32.8		2	98.2	5.6	31.4	87.5	33.3
	3	98.2	11.9	32.9	93.8	37.8		3	90.7	17.5	33.1	84.6	3.94
	4	94.5	25.4	35.6	91.2	46.1		**4**	**83.3**	**36.5**	**36.5**	**83.3**	**50.6**
	**5**	**85.2**	**43.7**	**39.8**	**87.1**	**56.1**		5	59.3	64.3	40.8	76.9	62.8
	6	81.5	62.7	48.4	88.5	68.3		6	31.5	85.7	47.1	73.3	69.4
	7	68.5	74.6	54.9	85.3	72.8		7	7.4	98.4	71.4	71.1	71.1
	8	51.9	84.1	55.1	78.6	74.4							
	9	25.9	92.1	58.3	73.7	72.2							
	10	5.6	99.2	80.0	70.9	71.1							
PAS	1	100.0	1.6	30.9	100.0	31.1	mPAS	1	100.0	2.4	31.1	100.0	31.1
	2	100.0	4.8	31.6	100.0	33.3		2	98.2	5.6	31.4	87.5	33.3
	3	98.2	12.7	33.5	94.7	38.3		3	94.5	17.5	34.0	88.9	40.6
	4	90.8	23.0	34.7	86.1	43.3		**4**	**87.0**	**30.2**	**36.1**	**85.1**	**47.2**
	5	90.8	38.9	39.7	90.7	54.4		5	77.8	50.8	41.2	82.1	58.9
	**6**	**81.5**	**56.4**	**44.4**	**86.4**	**63.9**		6	64.8	68.3	46.7	81.0	67.2
	7	68.5	72.2	50.7	83.2	71.1		7	29.6	84.9	44.1	72.6	68.3
	8	55.6	85.7	61.7	80.4	76.7		8	9.3	96.0	50.0	70.6	70.0
	9	25.9	92.1	56.0	73.6	72.2							
	10	7.4	96.8	50.0	70.4	70.0							

*AS* Alvarado Score, *mAS* modified Alvarado Score, *PAS* Pediatric Appendicitis Score, *mPAS* modified Pediatric Appendicitis Score, *NPV* Negative Predictive Value, *PPV* Positive Predictive Value

Cut-off values with their respective test characterstics have been highlighted for each of the appendicitis scores assessed

Table 5 outlines the test characteristics of the cutoff values for mAS, AS, mPAS, PAS and Lintula score (using original cutoff of 16). Kappa values for the PAS and mPAS, as well as the AS and mAS were 0.579 (0.467–0.691) and 0.597 (0.473–0.722) respectively.

**Table 5:** Comparison of test characteristics of cutoff values of AS, mAS, PAS, mPAS and LS

Score	Cut-off	# below cut-off with appendicitis	# above cut-off without appendicitis	Sensitivity	Specificity	PPV	NPV	Accuracy
Alvarado Score	5	8/63 (12.7 %)	71/117 (60.7 %)	85.2	43.7	39.3	87.3	56.1
Modified Alvarado Score	4	9/55 (16.4 %)	80125 (64.0 %)	83.3	36.5	36.0	83.6	50.6
Pediatric Appendicitis Score	6	10/81 (12.4 %)	55/99 (55.6 %)	81.5	56.4	44.4	87.7	63.3
Modified Pediatric Appendicitis Score	4	7/45 (15.6 %)	8/135 (65.2 %)	87.0	30.2	34.8	84.4	48.9
Lintula Score	16	22/124 (17.7 %)	24/56 (42.9 %)	59.3	80.1	57.1	82.3	73.2

*PPV* positive predictive value

*NPV* negative predictive value

## Discussion

In this study, we prospectively evaluated the test characteristics of pediatric appendicitis scores with and without laboratory investigations. We found that truncated versions of the AS and PAS which did not include bloodwork (mAS and mPAS) had a sensitivity and negative predictive value similar to the complete AS and PAS, albeit with a lower specificity. These modified scoring systems appear be as effective as the original scores in the discrimination between patients who are safe to be discharged with close follow-up versus those who need further investigation (i.e. bloodwork and/or diagnostic imaging) in the ED. In addition, we found that the Lintula Score had very poor sensitivity, limiting the score’s utility for capturing children with appendicitis within our population.

Our findings have clinical importance for the following reasons. First, the mAS and mPAS may be of significant use in primary care offices, walk-in clinics and urgent care facilities where laboratory investigations are not readily available. Children with a score <4 for the mPAS and mAS, may be safely sent home with close follow-up, while those above the cutoff would benefit from a referral for further evaluation in the ED (i.e. laboratory investigations, imaging studies and/or surgical consultation). Future studies to validate our results in the primary care setting are certainly warranted. Second, from a patient perspective, blood tests increase anxiety and pain. Given that our data demonstrate that a child with a mAS or mPAS of <4 has low probability of appendicitis, medical teams should consider not subjecting these low-risk children to a venipuncture, provided that adequate follow up is available. Using modified AS and PAS enables community physicians to forgo subjecting the child to a blood test, while maintaining quality of care. In terms of tertiary care applications, we have identified a low risk population (mAS and mPAS <4) in which the elimination of routine CBC may lead to significant improvements in ED process metrics (i.e. ED length of stay), though future implementation/ translational research studies are required. We recognize that the WBC count is an integral part of most ED pathways/protocols for evaluation of acute abdominal pain in children and that, in some cases, the patient will have blood work drawn prior to any physician assessment [20].

Our data suggests that children with mAS or mPAS ≥4 have an appreciable risk of pathology and likely require more definitive investigation through diagnostic imaging studies. Thus, in order to encourage timely delivery of appropriate care, ED physicians may not require the results of a WBC count prior to requesting diagnostic imaging as the suspicion of appendicitis is appreciable and imaging may be warranted regardless of a normal WBC count.

In this study, we specifically chose to focus on sensitivity and negative predictive value as the two most important test characteristics. Sensitivity was selected as a means of describing how well the score of 4 or greater identified all children that truly have appendicitis, while negative predictive value was chosen as a means to describe how well a score below 4 identified children without appendicitis. We acknowledge that, using cut-off values of 4 for in both mAS and mPAS, the specificity of the modified scores is lower than their original counterparts, which may result in a larger number of false positives. However, given that the objective of the scoring systems is to separate those who do from those who do not need further investigation, the balance between high sensitivity and negative predictive value versus lower specificity is acceptable.

Previous studies have evaluated the use of the WBC as a diagnostic tool in appendicitis [10–12]. Limitations of these studies may include 1) analyzing WBC as an independent, dichotomous variable for the diagnosis of appendicitis (i.e. not in combination with other clinical factors), 2) variation in the definition of leuckocytosis and 3) variations in the duration of symptoms at time of WBC testing. These studies have reported a wide range of test characteristics, with some studies reporting up to 40 % of children with pathology proven appendicitis having a “normal” WBC [10]. In addition, a significant number of children with negative appendectomy have been shown to have leukocytosis as most recently shown by Bates et al. (11 % neutophilia - differential ≥75 % neutrophils; 21 % leukocytosis - WBC >11500/μL), though lower cutoff values (<9 000 WBC/μL and < 8000 WBC/μL) significantly improved the accuracy of the WBC in predicting positive pathology in a population where clinical and imaging studies had suggested appendicitis [21]. Our study differed from the majority of the above studies in that our intent was not to directly evaluate the test characteristics of the WBC itself. Rather, we compared those of clinical scores commonly used in appendicitis with and without laboratory elements (WBC and neutrophil count). In our opinion, though the WBC lacks the test characteristics to be used as an independent predictor of appendicitis, the WBC may certainly be a valuable tool for the surgical team as a supportive test when clinical presentation (mAS or mPAS ≥4) suggests appendicitis.

Scoring systems for the identification of pediatric appendicitis have been studied for decades, dating to the 1980’s [4–6]. We included the PAS and AS in our evaluation due to their profile within the literature and frequent use in clinical settings. Our inclusion of the LS rested in it’s reliance on data obtained exclusively from the clinical history and exam. Multiple validation studies have evaluated these scores use across settings, patient populations and cut-off values [1, 14–19, 22–25]. This body of literature confirms a wide range of test characteristics and suggests that these scores should essentially be used as one tool within the diagnostic process rather than absolute diagnostic criteria. Our data supports the use of mPAS and mAS as a screening tool, recognizing that children with scores ≥4 warrant further evaluation through laboratory, imaging studies or surgical consultation.

### Limitations

The main limitation of this study is the inter-observer variability, as individual elements of the scores (i.e. physical exam) may not be reproducible. Although these are rather basic components of an abdominal examination, personal experience may significantly affect the examiner’s interpretation of the exam. Mandeville et al. showed some discrepancy in inter-observer scoring, with only 88 % agreement for the Alvarado score and 83.5 % for the PAS [16]. To mitigate this, only senior pediatric residents, PEM fellows and PEM staff physicians were eligible to complete the case report form, ensuring a certain level of experience and thus optimizing accuracy. Secondly, our results may not be generalizable to all settings, as the physicians participating had significant training in pediatric care. Future studies assessing the mAS and mPAS in the community setting are warranted, as the results of our study have the potential to significantly impact community practice. Finally, due to the operational structure of our research assistant program, we were limited to enrolling patients between 8 AM and midnight, which may have introduced a population bias.

## Conclusion

A modified Alvarado and PAS can be used to identify children at low risk of appendicitis who may be carefully observed at home without the use of laboratory investigation. Future prospective validation should be performed to confirm test characteristics, identify efficiencies in ED processes and cost analysis of implementing clinical scores without laboratory investigations. In addition, translation to primary care settings should be evaluated to identify test characteristics when performed by non-ED/non-surgical clinicians and to determine impact on referral patterns.

## Additional file

Additional file 1: Published test characteristics for the Alvarado score, the Pediatric appendicitis score and the Lintula score used to calculate the sample size for the current study. Published test characteristics for the Alvarado score, the Pediatric appendicitis score and the Lintula score used to calculate the sample size for the current study [1, 4, 14–19]. (DOCX 16 kb)

## Abbreviations

AS: Alvarado score
CT: Computed tomography
LS: Lintula score
mAS: Modified Alvarado score
mPAS: Modified pediatric appendicitis score
NC: Neutrophil count
NPV: Negative predictive value
PAS: Pediatric appendicitis score
PEM: Pediatric emergency medicine
PPV: Positive predictive value
US: Ultrasound
WBC: White blood cell count
