# Supplementary material for: Test characteristics of common appendicitis scores with and without laboratory investigations: a prospective observational study
Source: BMC Pediatr. 2016 Aug 30;16(1):147. doi: 10.1186/s12887-016-0687-6 (PMC5006246; doi:10.1186/s12887-016-0687-6)
Supplement: Additional file 1: — Published test characteristics for the Alvarado score, the Pediatric appendicitis score and the Lintula score used to calculate the sample size for the current study. Published test characteristics for the Alvarado score, the Pediatric appendicitis score and the Lintula score used to calculate the sample size for the current study [1, 4, 14–19]. (DOCX 16 kb) [file 12887_2016_687_MOESM1_ESM.docx]

**Additional file 1: Table S1 – Published test characteristics for the Alvarado score, the Pediatric appendicitis score and the Lintula score used to calculate the sample size for the current study.**

|  | Alvarado score | | | | Pediatric appendicitis score | | | | | Lintula score |
| --- | --- | --- | --- | --- | --- | --- | --- | --- | --- | --- |
|  | (Alvarado, 1986) [4] | (Bond, Tully, Chan, & Bradley, 1990) [14] | (Schneider, Kharbanda, & Bachur, 2007) [15] | (Mandeville, Pottker, Bulloch, & Liu, 2011) [16] | (Samuel, 2002) [5] | (Schneider, Kharbanda, & Bachur, 2007) [15] | (Bhatt, Joseph, Ducharme, Dougherty, & McGillivray, 2009) [17] | (Goldman, Carter, Stephens, Antoon, & Mounstephen, 2008) [18] | (Mandeville, Pottker, Bulloch, & Liu, 2011) [16] | (Lintula, Kokki, Kettunen, & Eskelinen, 2009) [19] |
| **Type of study** | Retrospective | Prospective | Prospective | Prospective | Prospective | Prospective | Prospective | Prospective | Prospective | Randomized control trial |
| **N** | 305 | 187 | 588 | 287 | 1170 | 588 | 246 | 849 | 287 | 126 |
| **Age** | 4-80 yrs | 2-17 yrs | 3-21 yrs | 4-17 yrs | 4-15 yrs | 3-21 yrs | 4-18 yrs | 1-17 yrs | 4-17 yrs | 4-15 yrs |
| **Cutoff** | ≥ 7 | ≥ 7 | ≥ 7 | ≥ 7 | ≥ 6 | ≥ 6 | ≥ 6 | ≥ 6 | ≥ 6 | ≥21 |
| **Sensitivity** | 81%* | 90% | 72% | 76% | 100% | 82% | 93% | 72%* | 88% | 100% |
| **Specificity** | 74%* | 72% | 81% | 72% | 92% | 65% | 69% | 94%* | 50% | 88% |
| **PPV** | 93%* | 83%* | 65% | 76% | 96% | 54% | 61%* | 67%* | 67% | 83% |
| **NPV** | 46%* | 81%* | 85% | 72% | 99% | 88% | 95%* | 95%* | 79% | 100% |

* Calculation as published by Thompson (2012) [1]
